# Evaluation of Willingness to Accept COVID-19 Vaccine and Willingness to Pay among Pakistani Parents for Their Children Aged 5 to 11 Years: Findings and Implications

**DOI:** 10.4269/ajtmh.22-0363

**Published:** 2023-05-15

**Authors:** Khezar Hayat, Muhammad Farooq Umer, Hasan Mujtaba, Ayesha Babar Kawish, Muhamad Azam Tahir, Faiz Ullah Khan, Farman Ullah Khan, Muhammad Omer Iqbal, Talib Hussain, Anees Ur Rehman, Yu Fang

**Affiliations:** ^1^ Department of Pharmacy Administration and Clinical Pharmacy, School of Pharmacy, Xi'an Jiaotong University, Xi'an, China;; ^2^ Center for Drug Safety and Policy Research, Xi'an Jiaotong University, Xi'an, China;; ^3^ Shaanxi Center for Health Reform and Development Research, Xi'an, China;; ^4^ Research Institute for Drug Safety and Monitoring, Institute of Pharmaceutical Science and Technology, Western China Science and Technology Innovation Harbor, Xi'an, China;; ^5^ Institute of Pharmaceutical Sciences, University of Veterinary and Animal Sciences, Lahore, Pakistan;; ^6^ Department of Preventive Dentistry, College of Dentistry, Al-Ahsa, Saudi Arabia;; ^7^ Department of Oral Pathology, Shaheed Zulfiqar Ali Bhutto Medical University, Islamabad, Pakistan;; ^8^ Department of Public Health, Alshifa School of Public Health, Rawalpindi, Pakistan;; ^9^ Khalid Mehmood Institute of Medical Sciences, Sialkot, Pakistan;; ^10^ Shandong Provincial Key Laboratory of Glycoscience and Glycoengineering, School of Medicine and Pharmacy, Ocean University of China, Qingdao, China;; ^11^ Department of Pharmacy Practice, Faculty of Pharmacy, Bahauddin Zakariya, Multan, Pakistan

## Abstract

Vaccines are the most efficient and cost-effective tool to halt the transmission and prevention of COVID-19. The current study examined the willingness of parents to vaccinate their children against COVID-19. This was a cross-sectional study that used a questionnaire based on the Health Belief Model, previous history of COVID-19, willingness to accept, and willingness to pay for the COVID-19 vaccine. The questionnaire was administered among parents of children aged 5 to 11 years. Descriptive statistics, χ^2^ tests, and regression analysis were carried out for data analysis. A total of 474 respondents participated in this survey with a response rate of 67.7%. In our study, a majority of the respondents exhibited a willingness to accept the COVID-19 vaccine for their children (Definitely yes/Probably yes = 252, 53.2%); nevertheless, 229 (48.3%) respondents were unwilling to pay for it. More than three-quarters of the respondents were worried about the probability of COVID-19 infection in their children (*n* = 361, 76.2%) and were afraid of COVID-19-associated complications (*n* = 391, 82.5%). Likewise, most respondents showed their concerns regarding the effectiveness of the vaccine (*n* = 351, 74.1%), vaccine safety (*n* = 351, 74.1%), and the halal nature of the vaccine (*n* = 309, 65.2%). Respondents who were aged 40 to 50 years (odds ratio [OR]: 0.101, 95% CI: 0.38–0.268; *P* < 0.001), family income > 50,000 PKR (OR: 0.680, 95% CI: 0.321–1.442; *P* = 0.012), and location (OR: 0.324, 95% CI: 0.167–0.628; *P* = 0.001) were the factors that were likely to impact vaccine acceptance among parents. Education-based interventions are urgently required to improve COVID-19 vaccination acceptance among parents for their children.

## BACKGROUND

Years of extensive research in basic medical science led to an unprecedented expeditious response to the rare pneumonia-like symptoms now termed COVID-19, caused by a SARS-CoV-2 zoonotic spillover in the Wuhan seafood market. The virus was identified and genetically sequenced, and the race for vaccine development began. The unprecedented response based on years of basic medical research, regarding detection, genetic sequencing, and vaccine development to curb the COVID-19 pandemic was incredible.^1^ The pandemic catastrophe is greater than what was first predicted in early literature,^2^^,^^3^ causing havoc across the globe due to mortality, morbidity, and psychosocial and economic distress.^4^ The SARS-COV-2 genome has the potential to have point mutation, recombination, insertions, and deletions at 1 × 10^3^ per year.^5^ Circulating SARS-COV-2 genetic lineages are continuously evolving and emerging. These variants are categorized into four groups, of which delta is the variant of concern; other types are included in the variant being monitored, whereas no type to date has been categorized as a variant of high consequence. These continuous mutating variants have the potential to become divergent similar to influenza and can also mutate into a variant of high consequence that could be devastating.^5^ Pakistan has reported more than 1.57 million cases of COVID-19 and 30,635 deaths as of 20 December 2022.^6^

Vaccines are the most efficient and cost-effective tool to halt the transmission of the virus.^7^ Hence, resolution for extensive immunization globally was issued by the World Health Assembly in May 2020. All the emergency-approved vaccines by the WHO offer protection against circulating variants of SARS-COV-2 and should be administered to achieve herd immunity. Vaccines elicit an innate immune response, and the path to normalcy is mass immunization as quickly as possible.^5^^,^^7^^,^^8^ These vaccines were initially prioritized in some countries for adults because complications and mortality of COVID-19 in elderly people were high, but now they are being rolled out for adolescents and children. The major hindrance posing a serious threat to the global population for the mass coverage of the entire population in all the countries is vaccine hesitancy, a global phenomenon recognized by WHO as a top 10 health risk.^9^^,^^10^ Pakistan has a fragile healthcare system and compromised suboptimal vaccine coverage.^11^ Numerous factors are related to hesitancy and include literacy, knowledge, profession, perceived risk, trust in the healthcare system, and country of origin of the vaccine.^7^^,^^12^^–^^15^

As of February 2022, vaccination coverage in Pakistan for SARS-CoV-2 is only 26% despite an initial survey indicating that 56% of the respondents showed the willingness to get vaccination^7^ (https://ourworldindata.org/covid-vaccinations). To achieve herd immunity, vaccination coverage of 75% to 90% is required based on R_O_ COVID-19.^16^ Full coverage to curtail the virus requires planning intervention and measures at the general public level by health authorities, awareness, and assurance of safety and benefits.^17^^,^^18^ A continuous communication campaign is a must to build and strengthen trust in the health system, especially when the vaccine rollout is taking place for adolescents and children.^19^^–^^22^ Conversely, although COVID-19 vaccines induce immunogenicity, it is critical to establish the safety of these vaccines, especially when administered in a vulnerable group, given that in 2021, a few cases of myocarditis were reported in adolescents after vaccination.^21^^,^^23^ Parents/guardians of minor children are the sole decision-makers. To achieve high vaccine coverage, evaluation of risk perception and COVID-19 vaccine hesitancy in Pakistani children and their parents/guardians needs to be undertaken. The reported risk of complications in children for COVID-19 is less than in adults, although it can still cause serious disease and mortality.^24^ No vaccine was offered in Pakistan for children aged 5 to 11 years from the first week of August to the last week of September 2021. Sinopharm, a Chinese-origin vaccine, was the first to be administered to frontline healthcare workers in Pakistan. Later, other vaccines, such as Sinovac, AstraZeneca, Cansino, Sputnik, Pfizer-BioNTech, and Moderna, were also get registered for use in Pakistan.

It is imperative that before the vaccination is rolled out for children in Pakistan, evaluation of parents’ vaccine acceptance for their children aged 5 to 11 years, including parents’ willingness to pay for the vaccine, be performed. This will help the health authorities take specific measures to address parents’ concerns and understanding of the COVID-19 vaccine.

## MATERIALS AND METHODS

### Study design and settings.

A cross-sectional online survey study design was adopted for this study. This survey was self-administered and implemented through Google Forms. The snowball sampling technique and various social media platforms were used to disseminate the information/weblink for the questionnaire. Respondents living in Punjab province and Islamabad (the capital city of Pakistan) were approached; however, respondents from other regions were also included. Data were collected from the first week of August to the last week of September 2021.

### Study respondents.

Parents, both fathers, and mothers of children aged 5 to 11 years were our intended population for this study.

### Inclusion and exclusion criteria.

Respondents who were married have children aged 5 to 11 years and living in Pakistan (Punjab province and Islamabad) were included in this study, however, the respondents from other regions of Pakistan were not included.

### Study tool.

We designed a study tool to evaluate risk perception regarding the COVID-19 vaccine and hesitancy in its acceptance. The WHO in 2002 termed risk as “a probability of an adverse outcome, or a factor that raises this probability.” Vaccine hesitancy refers to a delay in acceptance or refusal of vaccination despite the availability of vaccination services. Risk perception and vaccine hesitancy are complex phenomena and vary among different settings and population groups over time due to various personal, religious, political, and circumstantial factors. The risk perception and vaccine acceptability for children also varies for different diseases and for different vaccines and is seldom uniform across an entire population. The Health Belief Model (HBM) is a validated tool that has been used by researchers in the past to gain an insight of vaccine acceptance among different participants. The HBM is expedient in tailoring interventions based on behavior change and will help in initiating healthy behavior coupled with treating and preventing infectious diseases.^25^^,^^26^ We designed a tool based on the aforementioned definitions of risk, vaccine hesitancy, risk perception, and the HBM.

Two authors (K. H. and M. F.) formulated the initial draft of the tool based on other studies for the current research,^13^^,^^26^^–^^29^ and another three experts reviewed and edited it before conducting a pilot study. The tool was then tested with 10 parents, and minor changes were made to finalize the tool. The final draft was prepared in both English and Urdu (The national language of Pakistan) to make it understandable for the study population with the help of a bilingual expert who also performed backward and forward translations to confirm the validity of the information.

The designed questionnaire was divided into four sections including: 1) demographic data, 2) perceived health beliefs about COVID-19, 3) source of information about COVID-19 vaccines, and 4) willingness to accept COVID-19 vaccination. Demographic data collected comprised information on age; gender; family income; education; occupation; employment status; urban or rural area of residence; personal health status; presence or absence of any chronic disease; and the COVID-19 disease status of respondents, their families, and friends. The perceived health beliefs on COVID-19 section comprised 15 questions, the first six of which were on the likelihood and consequences of acquiring COVID-19 for respondents and their children, whereas the last nine questions probed hesitancy or acceptance of the vaccines for the disease. The responses in this section were recorded as agreements and disagreements by the respondents for themselves and their children. The third section gathered data on various information sources from the respondents about COVID-19 disease and vaccines. The last section on willingness to accept COVID-19 vaccination used a Likert scale to gather responses.

The internal consistency of the final tool was determined and ensured the validity of the questionnaire, Cronbach’s alpha was > 7.

### Sampling.

Raosoft, an online sample size calculator, was used to calculate the sample size for this study. Given a 95% CI, 5% margin of error, and 50% response distribution, we needed 384 respondents for this study. The convenience sampling method was selected for this study because it was the only feasible approach due to restrictions on movement during the COVID-19 pandemic.

### Data analysis.

We used frequencies and percentages to present continuous and categorical variables, respectively. Pearson’s χ^2^ test was implemented to compare respondents’ sociodemographic features with their COVID-19 vaccine hesitancy, willingness to pay, and HBM constructs. Logistic regression was also applied to determine the factors that could influence vaccine hesitancy and willingness to pay. Different variables including age group, location, residence, education, occupation, monthly household income, COVID-19 exposure, and history of chronic disease were used to perform subgroup analyzes. All analyses were performed using SPSS version 13.0 (SPSS Inc., Chicago, IL), and tests were considered significant at a level of < 0.05.

## RESULTS

### Demographics.

A total of 700 respondents were approached to complete this survey, and 474 filled out the survey questions for a response rate of 67.7%. The predominant population of this survey was male (*n* = 311, 65.6%), aged 20 to 30 years (*n* = 142, 30.0%), with a family income/salary > 50,000 PKR (*n* = 214, 45.1%), and had a tertiary level of education (*n* = 283, 59.7%). More than half of the total sampled population was included (*n* = 331, 69.8%); most of these respondents lived in an urban area (*n* = 396, 83.5%) and were in excellent health (*n* = 260, 54.9%) (Table 1). Of the total sample, 155 respondents (32.7%) were suffering from a chronic disorder, and 202 (42.6%) were infected with COVID-19. Furthermore, more than half of the family members (*N* = 278, 58.6%) and friends (*N* = 338, 71.3%) of the respondents had at some time been infected with COVID-19.

**Table 1:** Demographics variables of the respondents (*N* = 474)

Variables	Frequency	Percentage
Age, years		
< 20	118	24.9
20–30	142	30.0
31–40	131	27.6
41–50	62	13.1
> 50	21	4.4
Gender		
Male	311	65.6
Female	163	34.4
Family income/salary (PKR)		
< 25,000	135	28.5
25,000–50,000	125	26.4
> 50,000	214	45.1
Education		
Primary or below	36	7.6
Secondary	155	32.7
Tertiary	283	59.7
Occupation		
Used	331	69.8
Student	88	18.6
Unemployed	55	11.6
Residence		
Urban	396	83.5
Rural	78	16.5
Health status		
Excellent	260	54.9
Good	191	40.3
Poor	23	4.9
Location		
Punjab	295	62.2
Islamabad	71	15.0
Others	108	22.8
Are you suffering from any chronic disease		
Yes	155	32.7
No	319	67.3
Have you been infected with COVID-19?		
Yes	202	42.6
No	272	57.4
Have family members had COVID-19?		
Yes	278	58.6
No	196	41.4
Friends with COVID-19 Infection		
Yes	338	71.3
No	136	28.7
Intent to receive COVID-19 vaccine		
Definitely yes/probably yes	252	53.2
Definitely no/probably not	222	46.8

PKR = Pakistanian rupees.

### COVID-19–related health beliefs.

Most of the respondents agreed with the perceived susceptibility of catching COVID-19 infection for their children (Table 2). For example, more than three-quarters of the respondents were worried about the likelihood of COVID-19 infection in their children (*n* = 361, 76.2%). Our respondents were also afraid of the complications of COVID-19 infection (*n* = 391, 82.5%) and believed that their children’s health would deteriorate if they contracted the disease (*n* = 349, 73.6%). Most of the respondents in our study believed that vaccination could prevent the risk of contracting COVID-19 and its complications for children (*n* = 397, 83.8%). However, more than three-quarters of the respondents noted their concerns regarding the effectiveness of the vaccine (*n* = 351, 74.1%), vaccine safety (*n* = 351, 74.1%), and the halal nature of the vaccine (*n* = 309, 65.2%). A significant proportion of the respondents of our study agreed that the vaccine could be administered to their children if they had complete information about it (*n* = 402, 84.8%), was received by many people (*n* = 355, 74.9%), and its safety was ensured (*n* = 404, 85.2%).

**Table 2:** Health Belief Model constructs

Variables	Agree		Disagree	
	Frequency	Percent	Frequency	Percent
Perceived susceptibility				
Chance of getting COVID-19 in the future is very high	316	66.7	158	33.3
I worry about the likelihood of getting COVID 19 for my children	361	76.2	113	23.8
Getting COVID-19 for my children is currently a possibility for me	357	75.3	117	24.7
Perceived severity				
Complications from COVID-19 are serious	391	82.5	83	17.5
Children will be very sick if they get COVID-19	349	73.6	125	26.4
I am afraid of getting COVID-19	367	77.4	107	22.6
Perceived benefits				
Vaccination is a good idea because it makes me feel less worried about COVID-19 infection in my children	407	85.9	67	14.1
Vaccination decreases chance of getting COVID-19 or its complications in my children	397	83.8	77	16.2
Perceived barriers				
I am concerned about the efficacy of the vaccination available	351	74.1	123	25.9
I am concerned about the safety/side effects of the vaccination available	351	74.1	123	25.9
I am concerned about the halal nature of the vaccination available	309	65.2	165	34.8
I am concerned about a faulty/fake COVID-19 vaccine	348	73.4	126	26.6
Cues to action				
Children will get vaccine after I receive complete information	402	84.8	72	15.2
Children will get vaccine if it is received by many in the public	355	74.9	119	25.1
Child will get vaccine if it does not cause undue side effects to vaccinated people	404	85.2	70	14.8

### Willingness to accept and willingness to pay for the COVID-19 vaccine.

**Figure 1.**
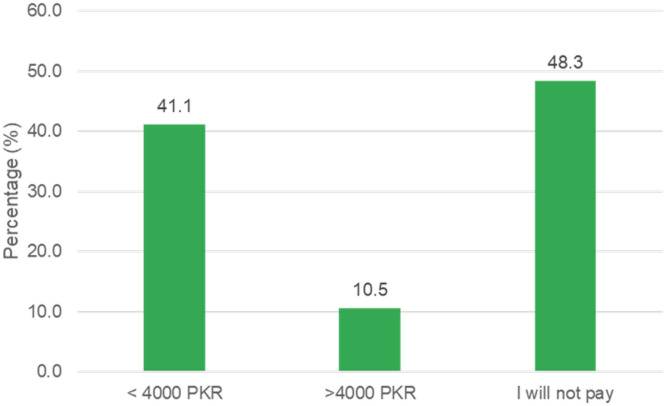
Willingness to pay for COVID-19 vaccine.

**Figure 2.**
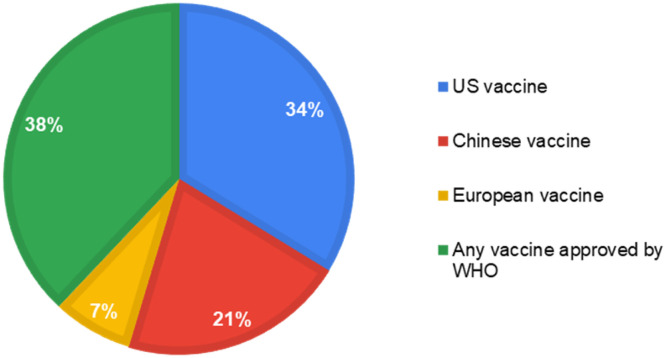
Preference of vaccine for COVID-19.

Most of our study respondents showed a willingness to accept (WTA) the COVID-19 vaccine for their children (Definitely yes/Probably yes = 252, 53.2%) nonetheless; 229 (48.3%) respondents were not in favor of paying for it (Figure 1). Supplemental Table 1 highlights the association between demographic variables with a willingness to accept and paying for the vaccine. It was found that most demographic variables, including age, gender, education, residence, occupation, health status, location, and previous COVID-19 experience, had a significant association with WTA and willingness to pay (WTP; < 0.05). Furthermore, the association between the HBM construct and WTA/WTP revealed a significant association at various constructs (< 0.05) as highlighted in Supplemental Table 2. One hundred eight respondents (38.0%) said that they would give any vaccination to their children that had been approved (Figure 2). A large number of respondents were getting COVID-19–related information from television/newspaper (*n* = 398, 84.0%), the official WHO website (*n* = 394, 83.1%), and social media (*n* = 382, 80.8%; see Supplemental Figure 1).

Multivariate logistic regression analysis revealed that age 40 to 50 years (odds ratio [OR]: 0.101, 95% CI: 0.38–0.268; *P* < 0.001), family income > 50,000 PKR (OR: 0.680, 95% CI: 0.321–1.442; *P* = 0.012), and location (OR: 0.324, 95% CI: 0.167–0.628; *P* = 0.001) were more likely to give the vaccine to their children. Moreover, respondents who were aged over 50 years (OR: 7.881, 95% CI: 2.209–28.122; *P* = 0.001), living 188 in a rural area (OR: 2.184, 95% CI: 1.095–4.359; *P* = 0.027), without the chronic disease (OR: 2.057, 95% CI: 1.105–189 3.827); *P* = 0.023), and their friends are COVID-19 positive (OR: 2.263, 95% CI: 1.336–3.833; *P* < 0.002) are more likely to pay for COVID-19 vaccine to administer the vaccine to their children (Table 3).

**Table 3:** Multivariate logistic regression analysis with intention to get vaccination and willingness to pay

Variables	Willingness to accept vaccine			Willingness to pay for vaccine		
	OR	95% CI	*P* value	OR	95% CI	*P* value
Age						
< 20	1	1	1	1	1	1
20–30	0.370	0.164–0.831	**0.016**	4.059	1.793–9.189	**0.001**
31–40	0.275	0.116–0.651	**0.003**	3.980	1.683–9.409	**0.002**
41–50	0.101	0.38–0.268	**< 0.001**	2.740	1.086–6.913	**0.033**
> 50	0.198	0.055–0.716	**0.013**	7.881	2.209–28.122	**0.001**
Gender						
Male	1	1	1	1	1	1
Female	0.653	0.394–1.080	0.097	0.782	0.480–1.274	0.323
Family income (PKR)						
< 25,000	1	1	1	1	1	1
25,000–50,000	0.354	0.158–0.793	0.315	1.741	0.790–3.834	0.169
> 50,000	0.680	0.321–1.442	**0.012**	1.048	0.454–2.416	0.913
Education						
Primary or below	1	1	1	1	1	1
Secondary	1.526	0.604–03.852	0.371	0.440	0.164–1.181	0.103
Tertiary	0.515	0.203–1.311	0.164	0.510	0.190–1.374	0.183
Occupation						
Used	1	1	1	1	1	1
Student	0.696	0.368–1.317	0.265	1.969	1.033–3.752	0.040
Unemployed	0.740	0.350–1.563	0.430	0.923	0.459–1.855	0.821
Residence						
Urban	1	1	1	1	1	1
Rural	1.123	0.589–2.140	0.724	2.184	1.095–4.359	**0.027**
Health status						
Very good	1	1	1	1	1	1
Good	0.957	0.587–1.559	0.860	1.070	0.669–1.710	0.778
Poor	1.035	0.337–3.173	0.952	0.593	0.185–1.905	0.381
Location						
Punjab	1	1	1	1	1	1
Islamabad	0.704	0.381–1.300	0.262	1.079	0.596–1.954	0.801
Others	0.324	0.167–0.628	**0.001**	1.132	0.621–2.062	0.686
Chronic disease						
Yes	1	1	1	1	1	1
No	1.584	0.788–3.185	0.197	2.057	1.105–3.827	**0.023**
Infected with COVID-19						
Yes	1	1	1	1	1	1
No	0.741	0.424–1.295	0.293	1.382	0.821–2.327	0.223
Family members with COVID-19						
Yes	1	1	1	1	1	1
No	1.223	0.720–2.076	0.457	0.994	0.597–1.655	0.982
Friends with COVID-19 infection						
Yes	1	1	1	1	1	1
No	1.160	0.687–1.960	0.579	2.263	1.336–3.833	**0.002**

OR = odds ratio; PKR = Pakistanian rupees. Bolded *P* values indicate statistical significance.

## DISCUSSION

Vaccination is the most suitable interventional strategy to help eradicate the ongoing COVID-19 pandemic across the world; however, its success is primarily dependent on the population’s willingness to be vaccinated and demand for the vaccine. The evidence regarding the demand and willingness of parents of paying for the COVID-19 vaccine for their children in a resource-limited setting is lacking. In the current study, we determined the demand, intentions, and willingness of the parents regarding the COVID-19 vaccine using the HBM.

In our study, nearly half of the respondents (46.8%) demonstrated an unwillingness to accept COVID-19 for their children. Numerous studies conducted in different regions have shown a varied level of parental vaccine hesitancy and acceptance. For example, a study conducted in Taizhou, China, found vaccine hesitancy among 52.5% of the total surveyed population.^30^ According to a recent meta-analysis, the COVID-19 vaccine acceptance rate among parents and guardians varies from 21.6% to 91.4% in different regions and countries.^31^ The low level of COVID-19 vaccine acceptance in Pakistan could be due to conspiracy theories and doubts. This was highlighted in our previous study.^32^ Educational interventions targeted at parents, informed by the evidence of causes of hesitancy, should be investigated by the government of Pakistan to enhance vaccine acceptance among parents.

The vaccine acceptance was significantly higher in respondents who were male (54.4%) and had a tertiary level of education (76.6%). Similar studies conducted in Jordan, Kuwait, and Saudi Arabia have reported similar findings.^10^^,^^28^ For example, in a Saudi study, vaccine acceptance was higher among males (62.0%) and those who had a higher education (84.7%).^28^

Previously, the risk of vaccine hesitancy has been reported to be twice as high among people who do not have a history of comorbidities (OR: 1.95, 96% CI: 1.36–2.80).^33^ Similarly, in our study, respondents without chronic disease showed higher resistance to the COVID-19 vaccine for their children compared with those with chronic disease (53.6% versus 46.4%). This could be because the mortality rate associated with COVID-19 among children is low and only children who suffer from asthma, obesity, and sickle cell anemia with compromised immune systems fall into the vulnerable group.^34^ In addition, a low level of knowledge about the suitability of vaccines for children, lack of trust, and risk of possible vaccine-associated side effects may be the other factors that could enhance vaccine hesitancy among parents.^35^

As per previous studies,^36^^,^^37^ various HBM constructs in our study were also associated with the intention of getting the vaccine. For example, higher perception of benefits and susceptibility, as well as lower perceptions of barriers, in our study respondents were significantly correlated with the willingness to accept the vaccine.

Furthermore, in this study, the willingness of parents to pay for the vaccine was also analyzed. Nearly half of our respondents (51.7%) were willing to pay for the COVID-19 vaccine for their children. This is in agreement with previous studies.^29^^,^^36^ However, being a developing country, it is important to note that parents from many areas of Pakistan may be unable to afford this vaccine for their children. It has been demonstrated that parents’ willingness to pay for the vaccine is significantly associated with different constructs of HBM and demographic variables.^29^

Various sources are available to obtain up-to-date information on COVID-19 and vaccination. Most of our respondents used television/newspaper, websites of international organizations such as WHO, and social media. Previous studies have also indicated that people often use a variety of sources to obtain COVID-19-related information.^38^ For example, a study conducted in Turkey found television to be the main source of COVID-19 information (48.7%).^39^ Parents should be advised that there is misinformation about different aspects of COVID-19 that could have a negative influence on the acceptance of the vaccine.

There are a few limitations of this study that should be considered in policymaking. First, this study was conducted on a small sample and the results could have limited generalizability. Second, we have not recorded the age of the children in this study; however, studies have shown that the age of the children does not have any significant impact on vaccine acceptance. Third, the link to this survey tool was shared online with potential respondents, and the participation of those with low educational levels was limited, possibly due to a lack of Internet access. Fourth, self-reported metrics based on a smaller sample may be unable to predict the behavior of people in the future toward the COVID-19 vaccine. However, our study provides useful and valuable information on demand, intent, and willingness to pay for vaccines among parents, which could help to devise suitable policies to help improve vaccine uptake and acceptability.

## CONCLUSION

The study concluded that nearly half of the survey respondents demonstrated unwillingness to receive COVID-19 vaccination for their children aged 5 to 11 years. Likewise, the majority of the respondents refused to pay for the COVID-19 vaccination. Various HBM constructs had a significant impact on WTA and WTP for COVID-19 vaccination. Factors including older age (> 50 years), living in a rural area, not having a chronic disease, and those whose friends were diagnosed with COVID-19 were more likely to influence parental COVID-19 vaccination acceptance for their kids. Mass-level customized educational interventional approaches are needed to help improve perceived susceptibility and reduce perceived barriers among parents.

## Supplemental Material


Supplemental materials
