# Supplementary material for: Evaluation of Willingness to Accept COVID-19 Vaccine and Willingness to Pay among Pakistani Parents for Their Children Aged 5 to 11 Years: Findings and Implications
Source: Am J Trop Med Hyg. 2023 May 15;109(1):69–75. doi: 10.4269/ajtmh.22-0363 (PMC10323998; doi:10.4269/ajtmh.22-0363)
Supplement: Supplementary file 1 [file tpmd220363.SD1.pdf]

Supplementary file

T1. Association between demographics with willingness to accept and willingness to pay for COVID-19 vaccine

Variables	Willingness to accept vaccine			Willingness to pay for vaccine		
	Yes N=252	No N=222	p-value	Yes N=245	No N=229	p-value
**Age**						
less than 20 years	16 (6.3)	102 (45.9)	**<0.001**	98 (40.0)	20 (8.7)	**<0.001**
20-30 years	83 (32.9)	59 (26.6)		47 (19.2)	95 (41.5)	
31-40 years	88 (34.9)	43 (19.4)		59 (24.1)	72 (31.4)	
41-50 years	50 (19.8)	12 (5.4)		34 (13.9)	28 (12.2)	
>50 years	15 (6.0)	6 (2.7)		7 (2.9)	14 (6.1)	
**Gender**						
Male	137 (54.4)	174 (78.4)	**<0.001**	172 (70.2)	139 (60.7)	**0.029**
Female	115 (45.6)	48 (21.6)		73 (29.8)	90 (39.3)	
**Family Income**						
<25000	27 (10.7)	108 (48.6)	<0.001	103 (42.0)	32 (14.0)	<0.001
25000-50000	67 (26.6)	58 (26.1)		36 (14.7)	89 (38.9)	
>50000	158 (62.7)	56 (25.2)		106 (43.3)	108 (47.2)	
**Education**						
Primary or below	19 (7.5)	17 (7.7)	<0.001	10 (4.1)	26 (11.4)	<0.001
Secondary	40 (15.9)	115 (51.8)		109 (44.5)	46 (20.1)	
Tertiary	193 (76.6)	90 (40.5)		126 (51.4)	157 (68.6)	
**Occupation**						
Employed	162 (64.3)	169 (76.1)	0.006	196 (80.0)	135 (59.0)	<0.001
Student	51 (20.2)	37 (16.7)		25 (10.2)	63 (27.5)	
Unemployed	39 (15.5)	16 (7.2)		24 (9.8)	31 (13.5)	
**Residence**						
Urban	205 (81.3)	191 (86.0)	0.170	225 (91.8)	171 (74.7)	<0.001
Rural	47 (18.7)	31 (14.0)		20 (8.2)	58 (25.3)	
**Health status**						
Very Good	112 (44.4)	148 (66.7)	<0.001	158 (64.5)	102 (44.5)	<0.001
Good	126 (50.0)	65 (29.3)		78 (31.8)	113 (49.3)	
Poor	14 (5.6)	9 (4.1)		9 (3.7)	14 (6.1)	
**Location**						
Punjab	133 (52.8)	162 (73.0)	<0.001	177 (72.2)	118 (51.5)	<0.001
Islamabad	43 (17.1)	28 (12.6)		31 (12.7)	40 (17.5)	
Others	76 (30.2)	32 (14.4)		37 (15.1)	71 (31.0)	
**Chronic disease**						
Yes	52 (20.6)	103 (46.4)	**<0.001**	120 (49.0)	35 (15.3)	**<0.001**
No	200 (79.4)	119 (53.6)		125 (51.0)	194 (84.7)	
**Infected with COVID-19**						
Yes	80 (31.7)	122 (55.0)	**<0.001**	145 (59.2)	57 (24.9)	**<0.001**
No	172 (68.3)	100 (45.0)		100 (40.8)	172 (75.1)	
**Family members with COVID-19?**						
Yes	137 (54.4)	141 (63.5)	**0.044**	174 (71.0)	104 (45.4)	**<0.001**
No	115 (45.6)	81 (36.5)		71 (29.0)	125 (54.6)	
**Friends with COVID-19 Infection?**						
Yes	175 (69.4)	163 (73.4)	0.339	205 (83.7)	133 (58.1)	**<0.001**
No	77 (30.6)	59 (26.6)		40 (16.3)	96 (41.9)	

T 2. Association between health belief model construct with willingness to accept and willingness to pay for COVID-19 vaccine

Variables	Willingness to accept vaccine			Willingness to pay vaccine		
	Yes N=252	No N=222	p-value	Yes N=245	No N=229	p-value
**Perceived susceptibility**						
Chance of getting COVID-19 in the future is very high.						
Agree	163 (64.7)	153 (68.9)	0.239	190 (77.6)	126 (55.0)	**<0.001**
Disagree	89 (35.3)	69 (31.1)		55 (22.4)	103 (45.0)	
I worry about the likelihood of getting COVID 19 for my children.						
Agree	195 (77.4)	166 (74.8)	0.506	207 (84.5)	154 (67.2)	**<0.001**
Disagree	57 (22.6)	56 (25.2)		38 (15.5)	75 (32.8)	
Getting COVID-19 for my children is currently a possibility for me.						
Agree	192 (76.2)	165 (74.3)	0.638	213 (86.9)	144 (62.9)	**<0.001**

Disagree	60 (23.8)	57 (25.7)		32 (13.1)	85 (37.1)	
**Perceived severity**						
Complications from COVID-19 are serious.						
Agree	205 (81.3)	186 (83.8)	0.486	218 (89.0)	173 (75.5)	**<0.001**
Disagree	47 (18.7)	36 (16.2)		27 (11.0)	56 (24.5)	
Children will be very sick if they get COVID-19.						
Agree	175 (69.4)	174 (78.4)	**0.028**	190 (77.6)	159 (69.4)	**0.045**
Disagree	77 (30.6)	48 (21.6)		55 (22.4)	70 (30.6)	
I am afraid of getting COVID-19.						
Agree	202 (80.2)	165 (74.3)	0.129	207 (84.5)	160 (69.9)	**<0.001**
Disagree	50 (19.8)	57 (25.7)		38 (15.5)	69 (30.1)	
**Perceived benefits**						
Vaccination is a good idea because it makes me feel less worried about catching COVID-19.						
Agree	223 (88.5)	184 (82.9)	0.080	226 (92.2)	181 (79.0)	**<0.001**
Disagree	29 (11.5)	38 (17.1)		19 (7.8)	48 (21.0)	
Vaccination decreases my chance of getting COVID-19 or its complications.						
Agree	215 (85.3)	182 (82.0)	0.326	219 (89.4)	178 (77.7)	**0.001**
Disagree	37 (14.7)	40 (18.0)		26 (10.6)	51 (22.3)	
**Perceived barriers**						
I am Concerned about the efficacy of the vaccination available.						
Agree	169 (67.1)	182 (82.0)	**<0.001**	194 (79.2)	157 (68.6)	**0.008**
Disagree	83 (32.9)	40 (18.0)		51 (20.8)	72 (31.4)	
I am Concerned about the safety/side effects of the vaccination available.						
Agree	165 (65.5)	186 (83.8)	**<0.001**	188 (76.7)	163 (71.2)	0.168
Disagree	87 (34.5)	36 (16.2)		57 (23.3)	66 (28.8)	
I am Concerned about the halal nature of the vaccination available.						
Agree	130 (51.6)	179 (80.6)	**<0.001**	166 (67.8)	143 (62.4)	0.225
Disagree	122 (48.4)	43 (19.4)		79 (32.2)	86 (37.6)	
I am Concern about the faulty/fake COVID-19 vaccine.						
Agree	166 (65.9)	182 (82.0)	**<0.001**	180 (73.5)	168 (73.4)	0.979
Disagree	86 (34.1)	40 (18.0)		65 (26.5)	61 (26.6)	
**Cues to action**						
Children will get vaccine after I receive complete information.						
Agree	213 (84.5)	189 (85.1)	0.853	223 (91.0)	179 (78.2)	**<0.001**
Disagree	39 (15.5)	33 (14.9)		22 (9.0)	50 (21.8)	
Children will get vaccine if it is received by many in the public						
Agree	188 (74.6)	167 (75.2)	0.876	190 (77.6)	165 (72.1)	0.168
Disagree	64 (25.4)	55 (24.8)		55 (22.4)	64 (27.9)	
Child Will get vaccine if it does not cause undue problems to vaccinated people.						
Agree	212 (84.1)	192 (86.5)	0.470	221 (90.2)	183 (79.9)	**0.002**
Disagree	40 (15.9)	30 (13.5)		24 (9.8)	46 (20.1)	

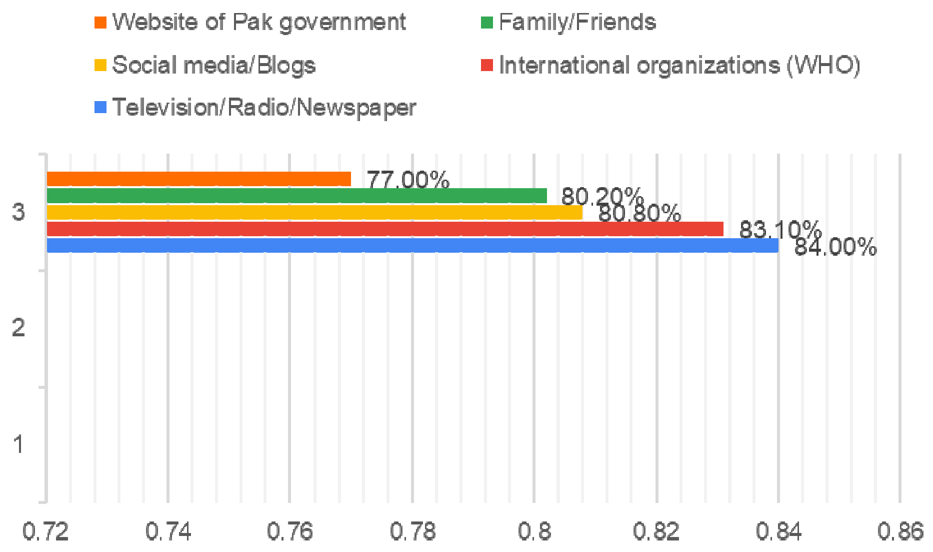

F 1. Sources of information related to COVID-19
